# Public perceptions of advance care planning (ACP) from an international perspective: a scoping review

**DOI:** 10.1186/s12904-023-01230-4

**Published:** 2023-07-28

**Authors:** Anne Canny, Bruce Mason, Kirsty Boyd

**Affiliations:** University of Edinburgh, Edinburgh, UK

**Keywords:** Advance care planning, Anticipatory care planning, Palliative care, Primary palliative care, Public opinion

## Abstract

**Background:**

Advance Care Planning (ACP) helps people discuss personal values, goals and priorities regarding future care with family and professionals. It can support care coordination and guide decision-making as health deteriorates. However, uptake remains low internationally. Poor communication and information due to Covid-19 pressures exacerbated public and professional criticism and concerns. Recent recommendations highlight the importance of understanding and addressing public perceptions about ACP combined with person-centred approaches to ACP conversations.

**Objectives:**

To explore public perceptions of ACP to inform increased public engagement and empowerment.

**Methods:**

Joanna Briggs Institute methodology was applied in a rapid scoping review. Three databases (Embase, MEDLINE, APA PsycInfo) were searched for English language reviews and primary or secondary research studies from 2015 to 2021. Following title and abstract review, two researchers screened full-text articles and performed data extraction independently using Covidence. Charted data were analysed for themes and subthemes starting with two recent published reviews. Emerging findings were added and data synthesis reviewed by the research team, including public-patient representatives, to achieve consensus.

**Results:**

Of 336 studies, 20 included reviews and research papers represented diverse public views, situations and contexts. Studies found poor public knowledge of ACP and widespread perceptions of confusing or accessible information. Multiple reports described little personal relevance, perceived risks of emotional distress, fears, mistrust and misconceptions about the purpose and scope of ACP. Studies identified public concerns stemming from reluctance to discuss death and dying despite this being just one aspect of ACP. Research with minority communities and marginalised groups found intensified concerns. Some studies cited people who valued maintaining autonomy by expressing their goals and preferences.

**Conclusions:**

Studies reviewed found many members of the public had negative or unclear perceptions of ACP. Improved knowledge and understanding appeared to influence perceptions of ACP but were not considered sufficient to change behaviours. The research provided valuable insights from members of the public that could inform current professional and societal debates about the future of ACP. Findings point to a need for novel approaches to ACP public information and involvement whilst bearing in mind societal norms, diverse cultures and contexts.

**Supplementary Information:**

The online version contains supplementary material available at 10.1186/s12904-023-01230-4.

## Background

Advance care planning (ACP) supports people of all ages with serious illnesses, deteriorating health from one or more long term condition or frailty in older age to think ahead and plan for what might happen [1]. Conversations between people and their clinicians or other care staff are central to ACP and often involve those close to the person [1, 2]. Talking about goals and values helps people prepare for decision making in the future. Preferences and recommendations for treatment and care can then be documented, shared and reviewed, as appropriate [3]. Assessment of ACP processes and outcomes evaluation are complex, but it is clear that uptake remains low despite evidence of benefits [4]. In the UK, Covid-19 placed extraordinary demands on healthcare services leading to prioritisation of aspects of ACP relating to hospital admission and cardiopulmonary resuscitation. However, some care planning practices led to public complaints, professional concerns and reduced support for ACP due to impersonal processes, insensitive communication, and poor public engagement [5–8]. Advance care planning continues to evolve internationally with calls for a broader approach that is more relevant and meaningful for people, families and wider society [9]. ACP processes should not over-emphasise documentation of end-of-life wishes that may evolve over time but rather accept inherent uncertainties around dying. Effective public engagement with ACP depends on understanding how people from diverse communities view planning ahead for changes in their health, and what information and support would be of most help to them [2].

Current debates around future directions for ACP in the USA and internationally concentrate on professional and policy perspectives [10]. Although ACP research includes patient and public perceptions, more attention needs to be paid to them in future ACP developments. Two recent reviews from 2020 provided valuable data which help explain some aspects of public perceptions of ACP. Grant et al., evaluated ACP alongside public views of hospice and palliative care, but their review was limited to studies from the USA and mostly comprised population based surveys [11]. Selman et al., conducted a rapid review of ACP evidence up to July 2020 which encompassed early Covid-19 articles [7]. Their comprehensive appraisal focused on wider aspects of ACP; individual, interpersonal, service provider, and system. We therefore designed a rapid scoping review on public perceptions of ACP. We defined ACP broadly as any type of future care planning for people with advanced illnesses, building on these two relevant reviews and including more recent articles. Our rapid scoping review aimed to synthesise peer reviewed literature that focused primarily on public perceptions of ACP with the research question: “What is the evidence to describe how members of the public perceive ACP that could inform wider public engagement and development of care planning for the future?”.

## Methods

A rapid scoping review is suitable for the description of existing literature on the topic of public perceptions about advance care planning and identify the extent of existing research evidence [12]. In a complex, multidimensional area of study like ACP, a scoping review facilitates the description of diverse published literature and can enhance understanding of multiple facets of public perceptions about ACP [13]. The review followed the Joanna Briggs Institute methodology for rapid scoping reviews based on a framework by Arksey and O’Malley with additional recommendations from Levac et al [13–15]. This ensured transparency and rigor in a process designed to provide a focused overview of evidence on public perceptions of ACP in the UK and internationally.

### Search strategy

A protocol for the search strategy and its conduct was drawn up by a project researcher (AC) in conjunction with an information specialist (MD) and piloted in January 2021. Following review by the project lead and other researcher (KB, BM) the protocol was revised. The revised search was run 16^th^ June 2021 by a project researcher (AC).

We used the OVID platform to search Embase, MEDLINE, and APA PsycInfo. As this was a rapid scoping review, studies were limited to those written in the English language due to time constraints and a lack of translation resources. We chose 1^st^ January 2016 to the present (at the time of the search) to enable us to focus on recent studies. Inclusion and exclusion criteria are shown in Table 1.

**Table 1:** Rapid scoping review public perceptions of ACP—inclusion and exclusion criteria

Inclusion criteria	Exclusion criteria
Articles of primary or secondary research focusing on public perceptions ^a^ of advance care planning ^b^ in any care setting	Studies only reporting professional perspectives
Qualitative, quantitative or mixed methods primary or secondary research in English that included public perceptions or experiences of ACP	Papers in a language other than English (as there were no resources for translation)
Trials, implementation studies, evaluations or audits of ACP if an element of that consisted of public perceptions of ACP prior to the intervention	Opinion papers, editorials, discussions and other non-research pieces. Conference papers, posters and abstracts. Research study protocols
Reviews of ACP if part of the review synthesised public perceptions of ACP	Research into ACP and ACP implementation studies focused solely on intervention outcomes

^a^Perceptions encompassed knowledge, understanding, beliefs, attitudes, and opinions about ACP

^b^ACP defined as any type of future care planning for people with any advanced progressive illness

**Fig. 1:**
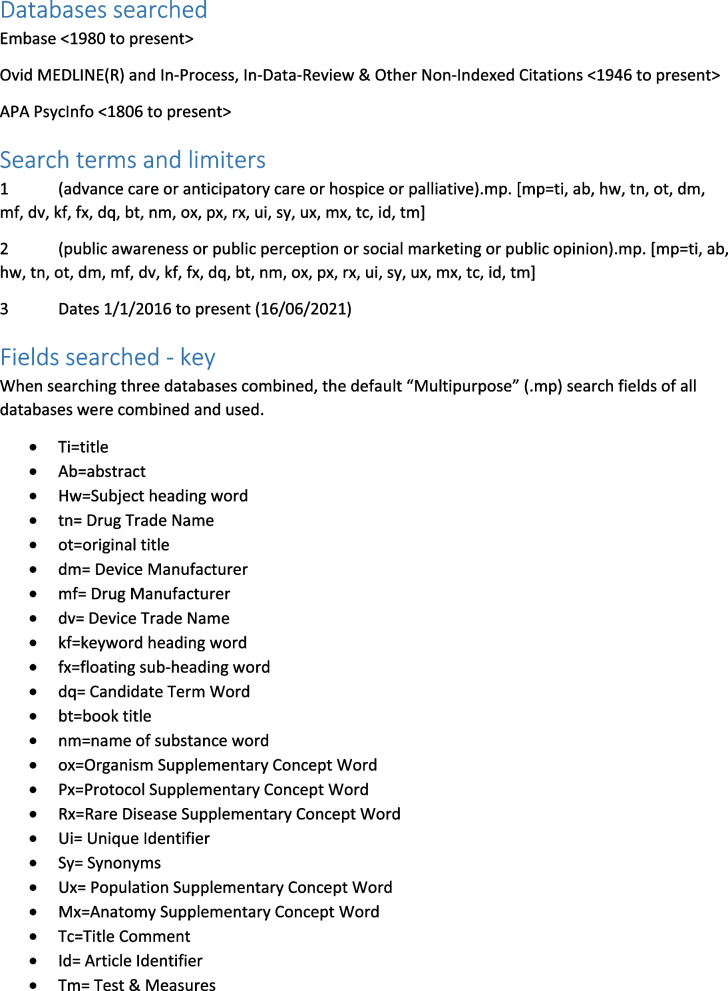
Ovid search details

The information specialist (MD) recommended a search strategy based on key words and inclusion of two additional text terms (hospice, palliative) alongside advance care planning. Anticipatory care planning is the preferred term in Scotland so was included in the search terms. Search terms were developed in line with the PCC framework (Population, Concept, Context) [13]. Concept terms searched were: advance care or anticipatory care or hospice or palliative. Context and Population terms were combined and defined as: public awareness or public perception or public opinion or social marketing. Studies could come from any country or care setting including home, care home, hospice or hospital. The search strategy, as performed on OVID, is presented in Fig. 1.

Search results were uploaded to Covidence by a project researcher (AC): an online platform that supports systematic data handling for literature reviews. [16] After checking and removal of duplicates, two researchers (AC, BM) conducted independent screening of article titles and abstracts then full text review before data extraction of relevant studies from the search results. Areas of conflict were resolved through discussions in online meetings (BM, AC). Members of the research team and project steering group were asked to suggest relevant, key articles published or in press through to June 2021. These were checked against the results and added if missing from the searches.

### Study selection process

**Fig. 2:**
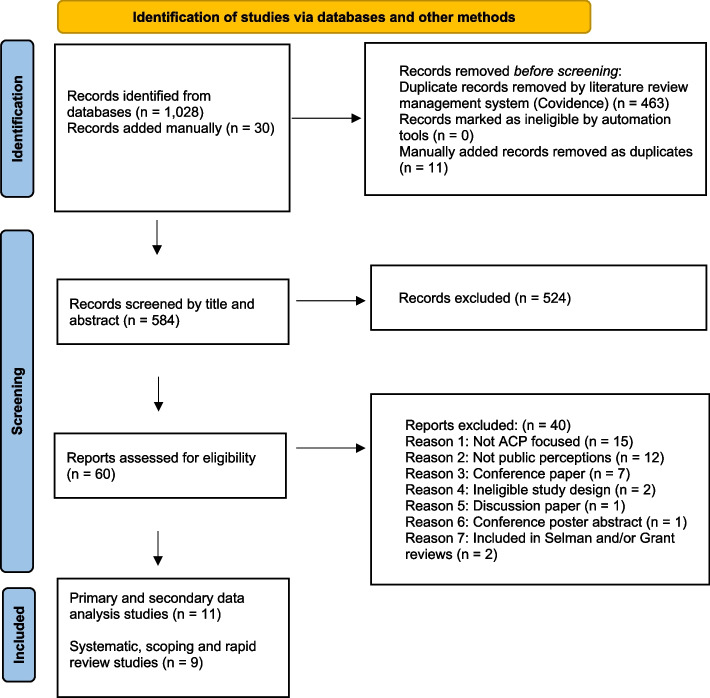
PRISMA 2020 flow diagram

Two researchers (AC, BM) read all the full text articles and agreed final selections guided by the inclusion and exclusion criteria (Table 1) and supported by a third-party reviewer (KB). Search results are shown in Fig. 2 in line with PRISMA-ScR guidelines [17–19]. One researcher (AC) extracted data from the included studies to an Excel spreadsheet that collated information on year of publication, study title, authors, country of origin and study design/methodology/population. This was reviewed by the second researcher (BM) and conflicts resolved through online-meetings. (Tables 2 and 3).

**Table 2:** Overview of nine systematic, scoping, and rapid reviews

Systematic, scoping and rapid reviews				
**Date published**	**Study title**	**Authors**	**Country of origin**	**Design/study population**
2015 February 13	Barriers to advance care planning at the end of life: An explanatory systematic review of implementation studies [20].	Lund S, Richardson A, May C	UK	Explanatory systematic review
2018 May 18	Overview of systematic reviews of advance care planning: Summary of evidence and global lessons [2].	Jimenez G, Tan WS, Virk AK, et al	Singapore/UK	Overview of systematic reviews
2018 June 29	Advance care planning: A systematic review about experiences of patients with a life-threatening or life-limiting illness [4].	Zwakman M, Jabbarian LJ, van Delden JJM et al	Netherlands	Systematic review
2018 September 18	Advance care planning for patients with chronic respiratory diseases: A systematic review of preferences and practices [21].	Jabbarian LJ, Zwakman M, van der Heide A	Netherlands	Systematic review
2020 March 10	Advance care planning for people living with dementia: An umbrella review of effectiveness and experiences [22].	Wendrich-van Dael A, Bunn F, Lynch J, et al	Belgium/UK	Evidence synthesis including systematic reviews and primary studies
2020 May 18	Public perceptions of advance care planning, palliative care, and hospice: A scoping review [11].	Grant MS, Back AL, Dettmar NS	USA	Scoping review encompassing over 9,800 study participants
2020 August 18	What enables or hinders people in the community to make or update advance care plans in the context of Covid-19, and how can those working in health and social care best support this process? [7].	Selman L, Lapwood S, Jones N, et al	UK	Rapid review synthesis
2020 September 21	Identifying barriers and facilitators to implementing advance care planning in prisons: A rapid literature review [23].	Macleod A, Nair D, Ilbahar E, et al	Australia	Rapid literature review
2021 February 3	Gypsy, Traveller and Roma experiences, views and needs in palliative and end of life care: A systematic literature review and narrative synthesis [24].	Dixon KC, Ferris R, Kuhn I, et al	UK	Systematic review and thematic analysis

**Table 3:** Overview of 11 primary and secondary data analysis studies

Primary and secondary data analysis studies				
**Date published**	**Study title**	**Authors**	**Country of origin**	**Design/study population**
2015 June 01	Relinquishing or taking control? Community perspectives on barriers and opportunities in advance care planning [22].	McLennan VE, Boddy JH, Daly MG, et al	Australia	Qualitative study, in-depth participant interviews (*n* = 26)
2015 October 04	What do Canadians think of advanced care planning? Findings from an online opinion poll [26].	Teixeira AA, Hanvey L, Tayler C, et al	Canada	Online opinion poll (*n* = 1,523)
2019 January 24	Dissonance on perceptions of end-of-life needs between health-care providers and members of the public: Quantitative cross-sectional surveys [27].	Cardona M, Lewis E, Shanmugam S, et al	Australia	Comparative study between views of healthcare workers and older people. (Doctors/ nurses; *n* = 360, members of the public *n* = 497)
2019 May 28	Framing advance care planning in Parkinson’s Disease: Patient and care partner perspectives [28].	Lum H, Jordan SR Brungardt A et al	USA	Qualitative descriptive study, (*n* = 60)
2019 December 12	“Whatever happens, happens” challenges of end-of-life communication from the perspective of older adults and family caregivers: A qualitative study [29].	Im J, Mak S, Upshur R, et al	Canada	Qualitative study of older adults with heart failure and their family caregivers, (*n* = 19)
2020 March 10	Lack of awareness and common misconceptions about palliative care among adults: Insights from a national survey [30].	Flieger SP, Chui K, Koch-Weser S	USA	Secondary data analysis of nationally representative self-reported data (*n* = 3,504)
2020 May 25	Exploring patient-reported barriers to advance care planning in family practice [31].	Bernard C, Tan A, Slaven M, et al	Canada	Multi-site cross-sectional study (*n* = 810)
2020 September 03	Associations between the intention to use early palliative care, sources of information, and attitudes toward a good death in Korean adults [32].	Kye SY, Han KT, Choi J, et al	Korea	Stratified nation-wide, cross-sectional study (*n* = 1,500)
2020 November 19	What is the public opinion of advance care planning within the Punjabi Sikh community? [33]	Landa, AS	UK	Survey, Sikh community (*n* = 311)
2021 March 19	Lean in, don’t step back: The views and experiences of patients and carers with severe mental illness and incurable physical conditions on palliative and end of life care [34].	Jerwood J, Ward G, Phimister D, et al	UK	Qualitative, semi-structured interviews (*n* = 8)
2021 May 17	‘It’s almost superstition: If I don’t think about it, it won’t happen’. Public knowledge and attitudes towards advance care planning: A sequential mixed methods study [35].	McIlfatrick S, Slater P, Bamidele O, et al	UK	A two phase, explanatory, sequential mixed-methods design (*n* = 1,201)

### Data analysis

We employed a simplified form of inductive coding to identify patterns in the data and aid interpretation [36]. After collating article details and content, reported themes and subthemes were categorised by the researchers (AC, BM) starting with findings from the two key review papers used to inform this study before moving on to the other review papers and finally each primary study. From these data, we generated domains covering key aspects of published research into public perceptions of advance care planning. Themes and domains were developed by a project researcher (AC) in the first instance before being reviewed by the full research team including our three Patient and Public Involvement (PPI) representatives, who are members of the public with a wide range of experiences as patients, carers and advocates.

## Results

Titles and abstracts of 336 articles (after removal of 402 duplicates) were screened. From these, 60 articles were included for full text review. After full text review, 40 records were excluded as they failed to meet the inclusion criteria (*n* = 38) or had been included in either of the 2020 scoping reviews (*n* = 2) (Fig. 2 – PRISMA diagram). Under 5% of articles required collective decision making. All the papers suggested by experts from the research team and steering group that met the inclusion criteria were present in the database searches.

A total of 20 studies met our inclusion criteria. They consisted of 9 reviews: systematic review (*n* = 6), rapid review (*n* = 2) and scoping review (*n* = 1), primary studies (*n* = 10), and secondary data analysis (*n* = 1). The eleven primary and secondary studies encompassed more than 9,400 participants. Studies originated from Europe (*n* = 9), North America (*n* = 6), Australasia (*n* = 3) and Asia (*n* = 2). Studies represented a combination of qualitative, quantitative and mixed-methods designs. They included exploration of ACP viewpoints from disease specific cohorts, ethnic minority populations, and vulnerable groups. The included studies are listed in Tables 2 (reviews) and 3 (primary and secondary research).

Findings of this rapid review are grouped into four domains: knowledge and engagement; fear, mistrust, and avoidance; misconceptions and misinformation; and public expectations of healthcare practitioners. See Tables 4 (reviews) and 5 (primary and secondary research) for a summary of key findings from the two sets of studies.

**Table 4:** Summary of key findings from nine systematic, scoping and rapid review studies

Year	Study title	Key Findings
2021	Gypsy, Traveller and Roma experiences, views and needs in palliative and end of life care: A systematic literature review and narrative synthesis [24].	Thirteen papers from eight studies were included in this systematic review. Variations between communities were apparent but three key themes were identified; 1. Strong family/community values. 2. Distinct health beliefs relating to superstitions around illness, personal care, death and bereavement. 3. Practical barriers to community healthcare; communication difficulties, limited awareness of ACP, low literacy skills and understanding of medical terminology, cultural tension and trust issues between patients and healthcare practitioners
2020	Identifying barriers and facilitators to implementing advance care planning in prisons: A rapid literature review [23].	This rapid review study reported low health literacy, limited knowledge of individual disease progression, access to personal medical information, treatment options and ACP in prison populations. For those who had knowledge, some expressed a lack of trust in the prison healthcare system and a lack of concern from prison healthcare practitioners and correctional officers. This led to many perceiving their ACP wishes would be ignored. Prisoners were also reluctant to commit to ACP for fear of being viewed weak or vulnerable by prison staff and inmates and at risk of being taken advantage of. These concerns influenced their willingness to engage in ACP conversations. Those who did engage in ACP processes expressed a sense of relief that their preferences were known
2020	What enables or hinders people in the community to make or update advance care plans in the context of Covid-19, and how can those working in health and social care best support this process? [7]	This rapid review found patients lacked knowledge about ACP. Some had poor health literacy and a perceived lack of access to tools. Patients expected GPs to initiate ACP discussions although much depended on interpersonal relationships. Many had negative emotions toward death and were reluctant to discuss end of life care, believing ACP was irrelevant. Those who were ready to talk preferred informal family discussions than formal documentation. Many believed that a post Covid-19 world would adversely impact access to ACP resources. Culture-related factors influenced the willingness to discuss or take up ACP within minority communities. Mistrust between patients and clinicians were documented particularly in a reluctance to discussing death
2020	Public perceptions of advance care planning, palliative care, and hospice: A scoping review [11].	This scoping review included 12 studies encompassing > 9,800 participants. The report found high awareness but low action-taking for ACP. A total of 80–90% of participants were aware of ACP, but only 10%-41% reported having named a healthcare proxy decision maker and 23–32% had completed a written directive. Proponents of ACP wished to protect their loved ones from the burden of future decision-making and associated complications after a patient death. Those who were disapproving did not trust health systems to follow their wishes. The report suggested the lack in uptake of this service was exacerbated by statutory regulations requiring notarised documents. Misconceptions included confusion between POLST (Physician Orders for Life-Sustaining Treatment) forms and ACDs/living wills which allow more nuanced goals to be documented
2020	Advance care planning for people living with dementia: An umbrella review of effectiveness and experiences [22].	This synthesis of systematic reviews and primary studies reported carer concerns of finding a balance between the timing of ACP conversations and patient cognitive ability to understand and make decisions. People with dementia were less distressed in discussing ACP than their more reluctant family members/carers who were often unaware of the ACP process. Positive family dynamics were instrumental in whether ACP conversations took place. The paper also reported trusting relationships between patients/family members/carers/healthcare practitioners as enablers of ACP, especially in the advance stages of dementia
2018	Advance care planning for patients with chronic respiratory diseases: A systematic review of preferences and practices [21].	ACP conversations were found to be uncommon in patients with respiratory diseases despite one study reporting 68% and 99% of patients with COPD and chronic lung diseases (respectively) willing to discuss care planning. Patients often held insufficient knowledge about the nature of their own disease and were ill-informed about ACP. Patients who were worried about becoming a burden on their family was found to be a facilitator for engagement in ACP. ACP was more acceptable to patients who had previously experienced loved ones having to decide about end-of-life care or who had experience of family death. The review found that patients perceived talking about ACP to be easier when they had reached an advanced disease stage
2018	Advance care planning: A systematic review about experiences of patients with a life-threatening or life-limiting illness [4].	This systematic review demonstrated many positive and negative patient opinions regarding ACP. Many patients felt ACP was helpful although participants found it difficult to engage in discussions around dying, particularly if they felt well. Patients were often not ready or open to talking about ACP for fear of distressing or burdening family members at an already stressful time. Those who did felt empowered and in control, feeling a sense of relief at reducing the burden on loved ones. Participants also mentioned that the ACP process offered an opportunity to think about their end of life care preferences
2018	Overview of systematic reviews of advance care planning: Summary of evidence and global lessons [2].	This overview of systematic reviews found ethnic and cultural differences impacted how the public perceived ACP. Caucasian members of the public were more likely to engage with ACP than ethnic minorities. Asian culture supported a collective, physician/family decision-making approach rather than appealing to the practice of self-determination and autonomy. The report highlighted a positive view towards ACP however, patients and caregivers avoided ACP conversations which were viewed as emotionally difficult. Patients and caregivers lacked readiness to engage in ACP especially when the nature of the illness was unpredictable. Older people, higher education level, and diagnosis of severe health conditions were associated with higher uptake of ACP
2015	Barriers to advance care planning at the end of life: An explanatory systematic review of implementation studies [20].	This explanatory systematic review reported perceived patient and family members’ uncertainty of prognostic/illness trajectory resulting in an unwillingness to engage in ACP. Strong behavioural norms encouraged avoidance actions around decisions to legitimise, record or share future care plans

**Table 5:** Summary of key findings from 11 primary and secondary data analysis articles

Year	Study title	Key Findings
2021	Lean in, don’t step back: The views and experiences of patients and carers with severe mental illness and incurable physical conditions on palliative and end of life care [34].	Participants described a complex history of mental health conditions coupled with a diagnosis of a terminal illness which adversely impacted their ability to engage with healthcare services. Feelings of mental health stigmatisation affected their willingness to try to access palliative care when they became incurably ill. Participants therefore, had little or no awareness of what support was available. Those participants who held knowledge felt a reluctance from clinical staff to initiate end of life or ACP conversations due to fears of de-stabilising individual patient’s mental health. This avoidance made participants feel invisible and abandoned. Most participants expressed a need for supportive or care networks to advocate on their behalf and for all healthcare staff, including mental health teams, to have better knowledge of what end of life/ACP services were available. Participants also felt healthcare staff needed develop confidence to talk to patients, ask questions and refer to appropriate well-coordinated services
2021	‘It’s almost superstition: If I don’t think about it, it won’t happen’. Public knowledge and attitudes towards advance care planning: A sequential mixed methods study [35].	This article found that 71.5% of participants (n = 1,201) had not heard of ACP. Of the 28.5% who knew the term, just 7% had engaged with ACP. ACP was associated with death and dying and considered a taboo subject for general discussion. Viewed as a ‘last resort’. Many misconceptions were aired from those who knew about ACP, i.e., involves medical care and treatment options only or only applicable at the end of life. When asked if participants would like to find out more about ACP, 68% said no. Most found the subject too difficult to broach with family members or healthcare practitioners for fear of causing upset or distress. However, 81.2% said they would be comforted knowing they had left wishes with their family. Participants were noted to state that if they did not think about their own mortality, it would not happen. On the other hand, some felt that if ACP was normalised, barriers surrounding the subject could be broken. The Covid-19 pandemic was viewed as a platform to create an acceptable rational for the public to discuss ACP
2020	What is the public opinion of advance care planning within the Punjabi Sikh community? [33].	Of 311 participants, just 30% held some understanding of what ACP meant and the same proportion knew how to access services. No specific cultural, religious or ideology barriers were found relating to discussing ACP with family and medical staff. Statements such as ‘too emotional to think about’ or ‘it’s a hard discussion to have’ were synonymous with other reports. The study found misconceptions around choosing/refusing life-sustaining treatments as 36% believed that CPR was mandatory as did 22% about intubation, ventilation and life support treatments. Results suggest a poor understanding of the decision-making processes and who can make final decisions
2020	Associations between the intention to use early palliative care, sources of information, and attitudes toward a good death in Korean adults [32].	Good family relationships were associated with low intention to use palliative care. Trust in medical staff, being involved in the decision-making process and being respected as an individual were associated with high intention to use palliative care. Patients were more inclined to trust healthcare practitioners than media reports. Asian culture associated palliative care with death and as such held negative views such as fear and distress towards discussing future care plans
2020	Exploring patient-reported barriers to advance care planning in family practice [31].	Participants lacked knowledge of ACP and felt others would take care of it. Those who knew about ACP did not see it as a priority or a necessity. The topic was viewed as too emotive, frightening, negative or depressing. Respondents were also concerned about causing conflict or distress to family members. Others did not trust family members to make care decisions. There was a clear indication that participants felt ACP discussions should be initiated by GPs
2020	Lack of awareness and common misconceptions about palliative care among adults: Insights from a national survey [30].	Less than one third of participants knew about palliative care. Female gender, having a college degree, higher income, and being a caregiver were associated with palliative care knowledge. 77% of participants held misconceptions around palliative care and 44% associated it with death. Misunderstandings were greater in those with less than a high school education and insurance status variables. Some participants noted that acceptance of palliative care meant ‘giving up’
2019	“Whatever happens, happens” challenges of end-of-life communication from the perspective of older adults and family caregivers: A qualitative study [29].	Patients tended to trivialise their illness with reports of ‘everything is fine’ and avoided discussing palliative care. Most preferred to hold a positive outlook and live day-to-day. They also reported a lack of control over events and did not see the point in having care planning discussions. Family members were not comfortable talking about care planning to healthcare practitioners who they did not know. Caregivers wanted and expected medical staff to provide ‘reality check’ in relation to palliative care
2019	Framing advance care planning in Parkinson Disease [28].	ACP was defined in a variety of ways by participants, largely shaped by their experience of Parkinson disease (PD) and other end of life experiences prior to diagnosis. These definitions influenced engagement in ACP. Some participants acknowledged the potential for future PD-related changes where constructive views of ACP led to planning trips or activities to achieve their goals of personal fulfilment and quality of life. Others considered ACP in relation to their perception of their illness and avoided ACP conversations, believing ACP primarily as end of life planning. Some patients preferred to live in the hope that their PD would be cured and denied the potential of death. Cognitive decline during disease progression and feeling overwhelmed by PD symptoms were a barrier to engaging in ACP. Lack of trust in health care systems was noted as some patients believed that regardless of a documented ACP, preferences and wishes would not be honoured. Incomplete knowledge, particularly regarding CPR led to the possibility of some patients making decisions they did not fully understand. Other misconceptions were identified in that some participants believed that completing advance directives only were sufficient which limited discussions about the wider process of ACP such as life values and the rationale or implication of the ACP document
2019	Dissonance on perceptions of end-of-life needs between health-care providers and members of the public: Quantitative cross-sectional surveys [27].	ACP directives were present for 23% of study participants, higher than the 14% previously reported Australia-wide. ACP conversations were considered informal by the general public rather than documented preferences. Most members of the public (92%) felt strongly about knowing the truth and wanted to be involved in treatment decisions. They agreed they should be informed by their GP about whether it is likely that they may not live more than a few months in contrast to clinicians’ perceptions
2015	What do Canadians think of advanced care planning? Findings from an online opinion poll [26].	Canadians had little knowledge of the term ACP. Participants tended to talk to family and friends about care planning rather than with healthcare practitioners. Older patients and women were more likely to consider ACP (75%) and more than 90% had discussed their preferences with family members. However, only 30% held discussions with doctors. Higher levels of education and income had a positive effect on the probability of knowing the term ACP and the recording of an ACP
2015	Relinquishing or taking control? Community perspectives on barriers and opportunities in advance care planning [22].	Participants lacked knowledge of ACP and held misunderstandings around the ACP process. Inaccessibility, complexity of forms, legal and financial confusion alongside fear and mistrust issues were noted by patients. Patients were reluctant to take action and ‘put-off’ ACP. Families were viewed as the driving factor behind ACP. Awareness of the importance of ACP increased with age

### Knowledge and engagement

Knowledge of ACP was described as low in all the review studies with authors reporting this as a key contributing factor behind poor uptake of ACP. Participants in many of the primary research projects were unaware of ACP before participating in the research study with cited quotations such as *‘I’ve never heard of them’* or *‘I didn’t know it existed’* and *‘Where does the man in the street get this information from? A lot of people go through life and have never heard of it’.* [25]. Dixon et al.reported inequitable access to palliative care services and opportunities for care planning among travelling communities and a lack of knowledge of what services were available [24]. Additionally, Jerwood et al.examined views and experiences of patients and carers with severe mental illness and incurable physical conditions; they found that all participants highlighted their own lack of knowledge of available services; *‘Actually, I don’t know what support is out there, it would be helpful for us to know, you know, what we can do is … even if it isn’t for now, so we know when we do need it*’. [34]. Even with good knowledge, engagement with ACP may be poor. Grant et al*.* found four studies in their review reporting high public awareness of ACP where 80–90% of people were informed but just 23–32% had started an ACP process. Familiarity with ACP concepts did not necessarily translate into active participation in ACP [11].

Several studies reported that increased knowledge and engagement with ACP was associated with older age, white ethnicity, female gender, a higher educational level and income, and being a healthcare professional [2, 26, 30]. Lack of easily accessible and straightforward information seemed to exacerbate poor knowledge and understanding and limited people’s ability to engage with ACP. This was evident in findings from an Australian interview study; *‘The complexity of the form is likely to be a significant factor in preventing people from completing the formal process, particularly as support with the process is limited or difficult to access’.* [25]. Having found evidence of inequities of access to ACP among informants, Selman et al.recommended provision of information and resources in other formats to help support informed decision-making about future care [7]. ‘*Video decisions aids and video and web-based ACP resources are particularly valuable. An important benefit is that these kinds of resources are effective among people with limited English proficiency, poor health literacy or from otherwise disadvantaged groups. Use written resources and ACP forms which are understandable, acceptable, sensitive, honest, and reliably capture patient wishes’.* [7].

One Canadian study found that although many participants reported having little knowledge of the term ACP, a substantial proportion were doing a form of ACP by having conversations or making decisions with their family and friends rather than with their doctor [26]. In their review, Selman et al.supported these inferences that some people preferred informal discussions with family members [7].

### Fear, mistrust, and avoidance

In the studies reviewed, researchers frequently identified fear, mistrust and avoidance as key factors behind a lack of engagement with ACP. McLennan et al., reported that patients feared they would be ‘*tempting fate*’ if they became more open to ACP conversations [22]. Other studies highlighted the finding that by recording an ACP, people thought they would lose their autonomy and independence. A commonly reported concern for many of the study participants related to mistrust of *‘what someone might do’*. [23, 25]. Several studies found that people believed their expressed preferences and wishes would not be carried out irrespective of having a documented ACP [23, 28]. In contrast, another study interviewed participants who said they were motivated to initiate ACP as a direct result of lacking trust in family members to make correct decisions on their behalf [31]. Mistrust was also identified in a rapid review of ACP in prisons by McLeod et al.; *‘Both prisoners and health practitioners described prisoner lack of trust in correctional health practitioners and/or saw the corrections system as barriers to engaging in ACP in prisons’*. [27]. Some studies highlighted previous negative healthcare experiences among participants as a driving force behind ACP avoidance, particularly with vulnerable groups and marginalised communities [24, 34]. For instance, Jerwood et al.recorded rich patient data from those with severe mental illnesses and a terminal illness; *‘Participants’ accounts were compounded by earlier experiences of prejudice and stigmatization when trying to access healthcare services’.* [34].

Many studies described ACP as being sad, depressing and too emotional or distressing for patients and families to engage with [22, 31, 33, 35]. Studies often acknowledged patients’ hesitancy in discussing or documenting future care plans. Many found participants reporting fear of the negative impact ACP might have on their family and GP and worries about being a burden, causing distress or altering family/physician dynamics [2, 4, 21, 33]. Conversely, some research cited proponents of ACP who stated that they wished to protect their close family and friends from the burden of future decision-making [11]. A study exploring ACP perceptions in patients with Parkinson disease found that some participants acknowledged the potential for future disease-related changes to their lives and their positive views of ACP led to planning trips or activities to achieve fulfilling personal goals and improved quality of life. However, a sense of hope and disavowal of future deterioration *‘prevented some patients and care partners from making concrete decisions about life-sustaining treatment or resuscitation’.* [28]. One review showed that patients with dementia expressed less distress about engaging in ACP conversations than their carers who often reported finding such decision-making stressful and challenging. Reluctance to start ACP was noted to be compounded by an uncertain disease trajectory and progressive loss of capacity [22].

### Misconceptions and misinformation

Many misconceptions which may hinder ACP processes were reported in these studies. For instance, terminology used by some studies conflicted with the overarching meaning and purpose of ACP found in international definitions. Grant’s review found that authors described ACP as *‘end of life planning’* in two out of four survey studies [11]. In their discussion, Grant et al. highlighted that *‘The public confuses ACP with end-of-life care’.* This was noted by McIlfatrick et al.who found that members of the public often viewed ACP as *‘a last resort when all treatment had failed’* or *‘care/treatment options once a terminal illness had been diagnosed’.* [35]. Similarly, Cardona et al.reported significant differences in public perceptions of an end-of-life time frame [27]. Almost half of respondents in this primary care study viewed end of life as the last days or hours before death. The authors suggested this short time-frame may have been responsible for lack of recognition of a need to discuss ACP earlier. Other misconceptions related to the age a person might begin ACP. Bernard et al.reported that people considered the process inappropriate due to perceiving that they were ‘too young’ with one of their participants noted to state: “I did not see this as necessary, it’s a bit soon. I am only 80” [31].

Even reasonable awareness of ACP may not overcome barriers if ACP processes are perceived to be difficult or are misunderstood. In two studies, public opinions of perceived ACP inaccessibility, cost, form complexity and length were viewed as significant factors in preventing participants from engaging with formal ACP [11]. A paper reporting survey data from members of the general public in 2019 found that people believed ACP conversations were merely informal rather than formally documented preferences [27]. A survey study of opinions of ACP within the Punjabi Sikh community in the UK (2020) noted misconceptions around cardiopulmonary resuscitation, intubation, ventilation and other life-support treatments; respondents believed they had no say in the decision making processes around these medical interventions [33].

### Public expectations of healthcare practitioners

Several studies reported patient expectations that healthcare staff should initiate ACP conversations [7, 20, 27, 31]. However, other research found beliefs that professionals lacked the time or inclination to offer ACP, leading patients not to raise the topic of ACP with them [31]. Many participants in another study (68%) felt it was the doctor’s duty to inform them of their life expectancy if they had a chronic illness, even if they did not ask [27].

## Discussion

We reviewed recent and current research reporting public perceptions of ACP to identify possible reasons for low uptake of ACP across the UK and internationally. Our findings, which are drawn from a wide range of studies with diverse groups of people in different countries and across care settings offer insights to guide conversations between clinicians, patients and families, and may inform approaches to increasing public knowledge, understanding and engagement with ACP.

Our review does not assess the quality of the included studies as scoping reviews do not undertake formal quality appraisal. It is limited to completed studies reported in English and published (either online or in paper form) at the time of the search. Grey literature was not included and the scope was kept narrow by using just three databases; this means that we may have missed some relevant reports.

Our review builds on two reviews of public perceptions of ACP from 2020. Grant et al.(scoping review) reported public opinion questionnaire findings from the USA [11]. Selman et al.(rapid review) included articles from an international perspective to July 2020 [7]. This study builds on that evidence. An emergent finding in this review was an increase in studies from diverse groups and marginalised communities including prisoners, travelling communities, as well as disease-specific studies and research among people with mental health illness.

Articles in this review encompassed a broad range of social situations, cultures and contexts, and included perspectives from different health and care systems internationally. Some people clearly did value ACP as a way of expressing their goals and preferences and sharing those with family and professionals [37–39]. Many studies confirmed persistent lack of knowledge, low awareness and ongoing confusion around ACP [40–43]. This included poor understanding about what ACP means, its purpose, components and processes that was compounded by limited knowledge of people’s underlying health conditions and wider health literacy problems [23–25, 31]. A perceived lack of access to suitable information was noted as a major contributing factor to low uptake in studies among many groups of people [23, 25, 33, 34]. Careful design of content and presentation are recommended to maximise audience attention, comprehension and engagement [11, 44]. However, as highlighted by one 2020 review, merely offering better constructed information in different formats may not address persistent barriers to active participation in ACP. Exploring how different patients, carers and the general public perceive ACP could be prioritised when refining or redesigning ACP processes and practices [45].

Emerging evidence from studies in this review indicated that mistrust of health systems and practitioners may be common among minority communities and likely requires multidimensional solutions based on finding common ground between minority communities and healthcare professionals as well as building a shared understanding of specific group needs [21, 32]. Many of the papers we reviewed recommended that this should be aligned with training for healthcare professionals who work within such communities to enhance their ability to offer culturally insightful palliative care and future care planning while respecting the values and preferences of individual families and patients [4, 21, 24, 28, 32].

A common barrier to ACP conversations reported in these studies and elsewhere is the perception that ACP is intended for people who are close to the end of their lives rather than being about future care planning more generally. It has been noted that discussions around end of life care, which is only one element of ACP, were often viewed as too difficult and emotionally distressing due to social taboos and the risks of causing family members and/or professionals distress that people still fear [46, 47]. Much research around public perceptions of ACP has focused on older people who are often seen as a priority group but we found studies where elderly people considered themselves as too young and fit for ACP [47, 48]. Perceptions of personal relevance have an important impact on people’s engagement with ACP [48]. More recently, there has been a shift in the scope of ACP to include individuals of all ages living with life-limiting conditions so that they too can be offered opportunities to become better informed about their health and care and participate actively in shared decision-making and planning ahead [44]. This is particularly important for people who lack decisional capacity due to their age or illness. ACP policy and practice in some parts of the UK now encourage this wider approach and active involvement of proxy decision-makers in personalised future care planning [49, 50]. Such initiatives may also help to normalise perceived stigma around discussing death, dying, loss and caring and lead to more open honest and constructive conversations [51].

Future care planning needs to account for the uncertain and often fluctuating nature of decision-making that occurs along the continuum of different serious illnesses, multi-morbidity and general frailty. For many people, deciding what care they may wish to have when dying was too difficult to consider, whereas decision-making in the present moment or near future was easier and more tangible [52]. An individualised, flexible, and repeatedly reviewed ACP process that supports patients and families/carers through their unique life journey would seem a more acceptable approach to future care planning. Emerging models of ACP can encompass wider personal values, goals and priorities, specific plans tailored to individual health and care situations, and recommendations for emergency treatment and care to guide professionals and proxy decision-makers.

## Conclusion

This review found that patients, carers and members of the public have many misconceptions in how they perceive ACP stemming from deeply held beliefs and values and not just from a lack of knowledge or due to confusing and inaccessible information. Many studies described lack of personal relevance, perceived risks of emotional distress, fears, mistrust and misconceptions about the purpose and scope of ACP. Research with minority communities and marginalised groups found intensified concerns. The studies included provide valuable insights about the perceptions of members of the public that could inform current professional and societal debates internationally about the future direction of ACP. Our review indicated that prevailing approaches to ACP may not be acceptable to many people. A redesign of ACP processes seems essential and timely given the growing numbers of people living with serious illness and declining health in the UK and internationally.

## Supplementary Information

**Additional file 1.**

## Data Availability

Original data is available upon reasonable request from the corresponding author: Dr Anne Canny.

## Abbreviations

ACD: Advance care directive
ACP: Advance/anticipatory care planning
CPR: Cardiopulmonary resuscitation
PCC: Population, Concept, Context
POLST: Physician orders for life sustaining treatment
